# Acupressure for anxiety: a pilot study of a nurse-led acupressure intervention for patients receiving chemotherapy

**DOI:** 10.1093/oncolo/oyag166

**Published:** 2026-04-30

**Authors:** Laura Rhee, Susanne Cutshall, Abbey Metzger, Margaret Kruger, Jenna Burckhard, Molly Mallory, Fran Whalen, Stacy D’Andre, Kathryn Ruddy, Charles L Loprinzi, Karen M Fischer, Jenn M Manggaard, Elizabeth Cathcart-Rake

**Affiliations:** Mayo Clinic, Division of Community, Internal Medicine, Geriatrics, and Palliative Care, 200 First St SW, Rochester, MN 55905, United States; Mayo Clinic, Division of Community, Internal Medicine, Geriatrics, and Palliative Care, 200 First St SW, Rochester, MN 55905, United States; Mayo Clinic, Division of Community, Internal Medicine, Geriatrics, and Palliative Care, 200 First St SW, Rochester, MN 55905, United States; Mayo Clinic, Department of Medical Oncology, 200 First St SW, Rochester, MN 55905, United States; Mayo Clinic, Department of Medical Oncology, 200 First St SW, Rochester, MN 55905, United States; Mayo Clinic, Division of Integrative Medicine, 200 First St SW, Rochester, MN 55905, United States; Mayo Clinic, Division of Community, Internal Medicine, Geriatrics, and Palliative Care, 200 First St SW, Rochester, MN 55905, United States; Mayo Clinic, Department of Medical Oncology, 200 First St SW, Rochester, MN 55905, United States; Mayo Clinic, Department of Medical Oncology, 200 First St SW, Rochester, MN 55905, United States; Mayo Clinic, Department of Medical Oncology, 200 First St SW, Rochester, MN 55905, United States; Mayo Clinic, Department of Medical Oncology, 200 First St SW, Rochester, MN 55905, United States

**Keywords:** acupressure, anxiety, chemotherapy

## Abstract

**Background:**

Anxiety is a common symptom experienced by patients with cancer, who are increasingly interested in non-pharmacologic symptom management. Acupressure, a noninvasive intervention thought to impact the neurohormonal axis, has shown promise in reducing anxiety. However, research on acupressure for anxiety in patients with cancer has been limited.

**Methods:**

Individuals receiving chemotherapy, who reported baseline anxiety, were eligible for this study. Consenting participants received one 15-minute nurse-led acupressure session and completed pre- and post-intervention surveys. The primary endpoint was a change in anxiety. Participants were also asked about symptoms of nausea, pain, well-being, relaxation, worry, and distress. Participants were invited to learn self-acupressure techniques. These participants were asked to complete a final survey 1-week post-intervention.

**Results:**

Thirty adult patients participated in this study. Mean baseline anxiety was 5 on a 0 to 10 Likert scale. Immediate post-intervention anxiety scores significantly improved for all thirty patients to an average of 2.0 (*P* < .001). Mean nausea scores improved from 1.5 at baseline to 0 post-intervention (*P* < .001), and pain scores improved from 2.0 at baseline to 1.0 post-intervention (*P* = .001). Participants reported significant improvement in sense of wellbeing (*P* = .0004), distress (*P* < .001), ability to achieve relaxation (*P* < .001), and ability to control their worries (*P* < .001). The improvement in anxiety, well-being, and relaxation was sustained at one week. 100% of participants stated that they would recommend acupressure for symptom management.

**Conclusion:**

Acupressure is a promising non-pharmacologic intervention for the management of cancer-associated anxiety.

Implications for Practice Acupressure offers a promising non-pharmacologic option for anxiety management in oncology settings. It is well tolerated and can be taught for self-administration, making it suitable for integration into routine care. The intervention’s benefits appear to extend beyond anxiety, improving other symptoms commonly experienced by cancer patients.

## Introduction

Anxiety is a prevalent and often undertreated symptom among patients with cancer, affecting over 20% of this population.^1^^,^^2^ The experience of anxiety contributes to additional symptoms, impaired treatment adherence, and diminished quality of life.^3^ While pharmacologic interventions exist, their use may be limited by contraindications, side effects, and patient preferences for non-pharmacologic approaches.^4^ Acupressure, a noninvasive technique rooted in Eastern Asian medicine, is thought to impact the neurohormonal axis by activating specific acupoints across meridians to restore balance of physiologic energy, or *Qi*. Contemporary research suggests that the meridians may represent the body’s fascial network and vessel nerve bundles, and stimulation may trigger electromagnetic inductive effects.^5^ Acupressure is accessible, cost-effective, and can be self-administered, making it an attractive option for patients seeking integrative therapies.^6^^,^^7^

## Methods

### Participants

Thirty adult patients undergoing cancer-directed infusion therapy who reported baseline anxiety (≥1 on a 0 = no anxiety to 10 = severe anxiety Likert scale) were enrolled. Eligibility required willingness to participate in a nurse-directed acupressure session. Consent and permission to touch were obtained verbally. Demographic and symptom surveys were administered before the intervention.

### Intervention

**Figure 1.**
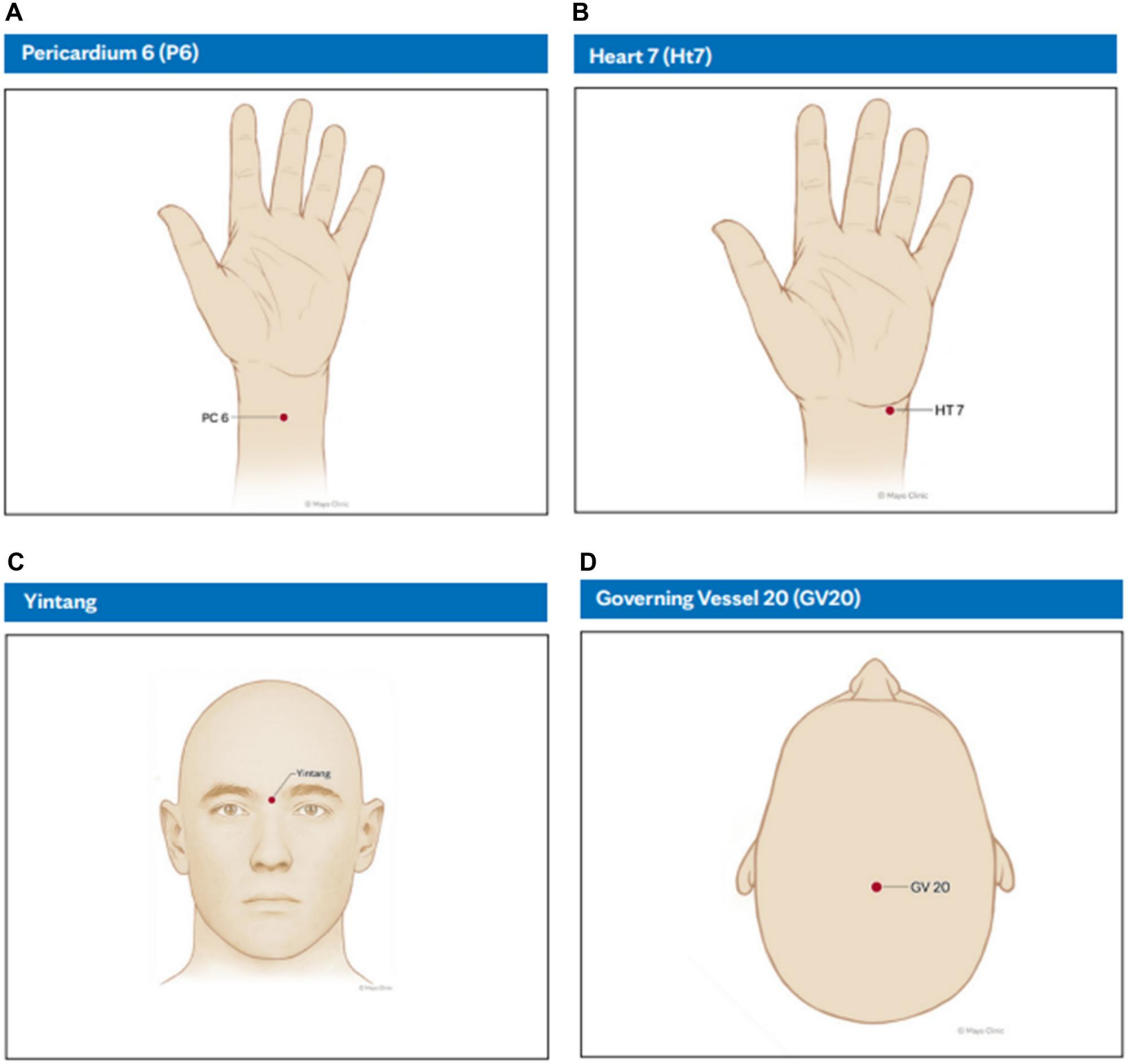
Acupressure point locations. (A) P6 (Pericardium 6), (B) HT7 (Shenmen), (C) Yintang, (D) GV20/DU20 (Baihui). Pressure is applied lightly with a gentle massage in a circular motion or held with steady pressure at a 90-degree angle; pressure is gently increased based on patient feedback. Pressure is held for 15-30 s up to 1-2 min based on patient tolerance and experience. All points were stimulated starting with the most superior point and proceeding inferiorly (GV20/DU20, YinTang, P6, HT7) unless the point was not deemed appropriate (points where the skin was broken, irritated, blistered, or bruised and areas with lines, tubes, or devices should be avoided).

Nurse-led acupressure sessions lasted 15 min and targeted specific acupoints: P6 (Pericardium 6), HT7 (Shenmen), Yintang, and GV20/DU20 (Baihui) (Figure 1). Pressure was applied using the thumb or pointer finger in a circular motion or with steady pressure, adjusting for patient comfort. Sessions avoided areas with broken skin or medical devices. After the intervention, participants completed post-session surveys and, if interested, received education on self-acupressure. Follow-up surveys and phone calls were conducted one week later to assess sustained effects and gather qualitative feedback (Figure S1).

### Measures

The primary endpoint was change in anxiety, measured on a 0-10 Likert scale. Secondary endpoints included changes in nausea, pain, well-being, relaxation, worry control, and distress. Surveys also captured medication use for anxiety. Statistical analysis used Wilcoxon signed-rank tests, with *P*-values <.05 considered significant.

## Results

### Demographics

The thirty patients enrolled in the study had a mean age of 56.5 years (SD 11.73; range 32–77); 76.7% were female, and 46.7% reported using medication for anxiety. The most common cancer types included breast (26.7%), hematologic (13.3%), melanoma (13.3%), pancreatic (10%), and others (Table S1).

### Nurse-led acupressure intervention: change in anxiety and other symptoms

Mean baseline anxiety was 5.0; the post-intervention score dropped to 2.0 (*P* < .001), with a median reduction of 2 points. Nausea improved from 1.5 to 0 (*P* < .001). Pain improved from 2.0 to 1.0 (*P* = .001). Well-being, relaxation, worry control, and distress all showed statistically significant improvements immediately post-intervention (Table 1).

**Table 1.** Difference between pre-test and post-test survey results.

	Pre (*N* = 30)	Post (*N* = 30)	Difference^a^ (*N* = 30)	*P*-value
Anxiety				<.001^b^
Median (IQR)	5.0 (3.0, 6.0)	2.0 (0.0, 4.0)	−2.0 (−4.0, −2.0)	
Nausea				<.001^b^
Median (IQR)	1.5 (0.0, 4.0)	0.0 (0.0, 2.0)	−0.5 (−1.0, 0.0)	
Pain				.001^b^
Median (IQR)	2.0 (0.0, 4.0)	1.0 (0.0, 2.0)	0.0 (−2.0, 0.0)	
Wellbeing				.0004^b^
Median (IQR)	4.0 (2.0, 5.0)	2.0 (0.0, 4.0)	−1.0 (−3.0, 0.0)	
Relaxing				<.001^b^
Median (IQR)	5.0 (3.0, 7.0)	2.0 (1.0, 3.0)	−3.0 (−4.0, −1.0)	
Worry/control				<.001^b^
Median (IQR)	5.0 (3.0, 6.0)	1.0 (1.0, 2.0)	−3.0 (−4.0, −1.0)	
Distressed				<.001^b^
Median (IQR)	4.5 (2.0, 6.0)	1.0 (0.0, 2.0)	−2.0 (−4.0, 0.0)	

a Differences were calculated as post-test–pre-test.

b Wilcoxon signed-rank test *P*-value.

### Self-led acupressure intervention: change in anxiety and other symptoms

Twenty-nine of the thirty participants received education in self-acupressure; follow-up survey completion rate was 31%. These participants reported a sustained improvement in anxiety, well-being, and relaxation after one week (Table 2). Nausea, worry control, and distress were slightly worse at follow-up compared to immediately post-intervention but remained improved from baseline.

**Table 2.** Comparison of pre, post and self-led acupressure intervention survey results.

	Pre (*N* = 30)	Post (*N* = 30)	Post2 (*N* = 11)
Anxiety			
Median	5.0 (3.0, 6.0)	2.0 (0.0, 4.0)	2.0 (1.0, 3.0)
Nausea			
Median	1.5 (0.0, 4.0)	0.0 (0.0, 2.0)	1.0 (0.0, 2.0)
Pain			
Median	2.0 (0.0, 4.0)	1.0 (0.0, 2.0)	0.0 (0.0, 2.0)
Wellbeing			
Median	4.0 (2.0, 5.0)	2.0 (0.0, 4.0)	2.0 (1.0, 5.0)
Relaxing			
Median	5.0 (3.0, 7.0)	2.0 (1.0, 3.0)	2.0 (0.0, 4.0)
Worry/Control			
Median	5.0 (3.0, 6.0)	1.0 (1.0, 2.0)	2.0 (1.0, 5.0)
Distressed			
Median	4.5 (2.0, 6.0)	1.0 (0.0, 2.0)	1.5 (0.0, 2.0)
Missing	0	0	1

### Participant perceptions of acupressure

The intervention was described as worthwhile and easy to perform. 100% of participants would recommend acupressure for symptom management (Table S2).

### Qualitative data

Follow-up calls indicated that participants found self-acupressure easy and beneficial for anxiety. Some requested further instruction for other symptoms, leading to referrals to integrative oncology or palliative care (Table S2).

## Discussion

Complementary management of cancer- and therapy-related symptoms is of interest to many patients due to concerns about drug toxicities, polypharmacy, and restrictions imposed by clinical trials and cancer therapy protocols pertaining to concomitant medication use.^8^ The lack of side effects in this study is consistent with the existing literature regarding acupressure.^9^

This pilot study supports that a nurse-led acupressure program focused on anxiety management for patients receiving cancer-directed infusion therapy was beneficial for the immediate improvement of anxiety. While this study utilized an 11-point Likert scale, as opposed to the Common Terminology Criteria for Adverse Events (CTCAE), making extrapolation challenging, the mean improvement in anxiety scores from 5 to 2 on the Likert scale may be similar to improvements from a grade 2 (limiting instrumental Activities of Daily Living, ADLs) to a grade 1 (mild symptoms; intervention not indicated) adverse event. Improvement in anxiety was sustained over one week for patients trained to perform ongoing self-acupressure.

The benefits of acupressure seem to extend beyond the management of anxiety. It was noted that nausea, pain, wellbeing, ability to achieve relaxation, and control of worry and distress were also improved immediately with the intervention, and improvements were sustained over time for patients performing self-acupressure. Improvements in nausea were not unexpected, as there is overlap in acupoints for anxiety and nausea. The improvement in pain scores may reflect the emotional aspects associated with pain beyond the physiologic level; pain and anxiety have a bidirectional relationship, so improvement in one may result in subjective improvement for both.^10^

The study’s limitations include its open-label, non-randomized design and potential bias from participant desire to please study staff. The presence of the nurse may have contributed to anxiety reduction, and survey responses could be influenced by social desirability. Nonetheless, the magnitude of improvement suggests a genuine therapeutic effect. The large improvements in anxiety that were seen in this study suggest that acupressure may be an innovative treatment strategy for patients with cancer. The improvement in anxiety is likely independent of baseline anxiety level, as patients with both mild and moderate baseline levels of anxiety noted improvement. In this study, acupressure was found to be beneficial for anxiety and other commonly associated symptoms, and the benefit appears to be sustained over time with continued practice of acupressure. Additional research is needed to understand if benefits persist beyond one week and if they exceed those of a placebo intervention. Therefore, a phase II sham-controlled prospective trial is underway to assess the effect of acupressure for the management of anxiety in patients with cancer. Acknowledgments: The authors acknowledge the cancer infusion center staff for their help in approaching patients to participate in this research and for their interest in providing integrative palliative care services to patients.

## Supplementary Material

oyag166_Supplementary_Data

## Data Availability

The data generated during the current study are available from the corresponding author on reasonable request.
