# Supplementary material for: Acupressure for anxiety: a pilot study of a nurse-led acupressure intervention for patients receiving chemotherapy
Source: Oncologist. 2026 Apr 30;31(8):oyag166. doi: 10.1093/oncolo/oyag166 (PMC13372679; doi:10.1093/oncolo/oyag166)
Supplement: oyag166_Supplementary_Data [file oyag166_supplementary_data.zip › Acupressure Pilot Supplemental Figure 1.docx]

Supplemental Figure S1. Study Schema

Final Survey

YES

Acupressure Teach

Baseline Survey

Enrollment of Eligible Participants

Interest in home acupressure?

Post-Intervention Survey

Acupressure Intervention

NO

Off Study
