# Supplementary material for: Acupressure for anxiety: a pilot study of a nurse-led acupressure intervention for patients receiving chemotherapy
Source: Oncologist. 2026 Apr 30;31(8):oyag166. doi: 10.1093/oncolo/oyag166 (PMC13372679; doi:10.1093/oncolo/oyag166)
Supplement: oyag166_Supplementary_Data [file oyag166_supplementary_data.zip › Acupressure Pilot Supplemental Table 1.docx]

Supplemental Table S1: Participant Demographic Information

|  | Total (N=30) |
| --- | --- |
| Age |  |
| Mean (SD) | 56.5 (11.73) |
| Median | 58.0 |
| Range | 32.0, 77.0 |
|  |  |
| Sex, n (%) |  |
| Female | 23 (76.7%) |
| Male | 7 (23.3%) |
|  |  |
| Primary Cancer, n (%) |  |
| Breast Cancer | 8 (26.7%) |
| Hematologic | 4 (13.3%) |
| Melanoma | 4 (13.3%) |
| Pancreatic | 3 (10.0%) |
| Cholangiocarcinoma | 2 (6.7%) |
| Colon | 1 (3.3%) |
| GE Junction | 1 (3.3%) |
| Fallopian tube | 1 (3.3%) |
| Gastric | 1 (3.3%) |
| Serous Ovarian | 1 (3.3%) |
| Small Bowel | 1 (3.3%) |
| Unknown primary | 1 (3.3%) |
| Vaginal | 1 (3.3%) |
| Urethral | 1 (3.3%) |
|  |  |
| Patient reported medication use for anxiety |  |
| Yes | 14 (46.7%) |
| No | 16 (53.3%) |
