# Supplementary material for: Acupressure for anxiety: a pilot study of a nurse-led acupressure intervention for patients receiving chemotherapy
Source: Oncologist. 2026 Apr 30;31(8):oyag166. doi: 10.1093/oncolo/oyag166 (PMC13372679; doi:10.1093/oncolo/oyag166)
Supplement: oyag166_Supplementary_Data [file oyag166_supplementary_data.zip › Acupressure Pilot Supplemental Table 2.docx]

Supplemental Table S2: Participant evaluation of acupressure intervention

|  | Total (N=30) |
| --- | --- |
| It was worthwhile to receive acupressure, n (%) |  |
| 3 | 1 (3.3%) |
| 6 | 1 (3.3%) |
| 7 | 2 (6.7%) |
| 8 | 4 (13.3%) |
| 9 | 5 (16.7%) |
| 10 (Strongly Agree) | 17 (56.7%) |
|  |  |
| I would recommend acupressure to others (family, friends, other patients), n (%) |  |
| 7 | 2 (6.7%) |
| 8 | 4 (13.3%) |
| 9 | 3 (10.0%) |
| 10 (Strongly Agree) | 21 (70.0%) |
|  |  |
| I would like to learn how to do acupressure so that I can repeat this at home, n (%) |  |
| 6 | 2 (6.7%) |
| 8 | 3 (10.0%) |
| 9 | 2 (6.7%) |
| 10 (Strongly Agree) | 23 (76.7%) |
